# Serum availability affects expression of common house-keeping genes in colon adenocarcinoma cell lines: implications for quantitative real-time PCR studies

**DOI:** 10.1007/s10616-016-9971-4

**Published:** 2016-06-23

**Authors:** Malgorzata Krzystek-Korpacka, Katarzyna Hotowy, Elzbieta Czapinska, Magdalena Podkowik, Jacek Bania, Andrzej Gamian, Iwona Bednarz-Misa

**Affiliations:** 1 Department of Medical Biochemistry, Wroclaw Medical University, ul. Chalubinskiego 10, 50-368 Wroclaw, Poland; 2 Department of Food Hygiene and Consumer Health, Wroclaw University of Environmental and Life Sciences, Wroclaw, Poland; 3 Wroclaw Research Center EIT+, Wroclaw, Poland

**Keywords:** Housekeeping genes (HKG), Reference genes, Serum starvation, Serum induction, geNorm, NormFinder

## Abstract

Careful selection of housekeeping genes (HKG) is prerequisite to yield sound qPCR results. HKG expression varies in response to hypoxia but the effect of manipulations of serum availability, a common experimental procedure, remains unknown. Also, no data on HKG expression stability across colon adenocarcinoma lines that would aid selection of normalizers suitable for studies involving several lines are available. Thus, we evaluated the effect of serum availability on the expression of commonly used HKG (*ACTB*, *B2M*, *GAPDH*, *GUSB*, *HPRT1*, *IPO8*, *MRPL19*, *PGK1*, *PPIA*, *RPLP0*, *RPS23*, *SDHA*, *TBP*, *UBC*, and *YWHAZ*) in seven colon adenocarcinoma cell lines (Caco-2, DLD-1, HCT116, HT29, Lovo, SW480, and SW620). Sets of stably expressed line-specific and pan-line HKG were validated against absolutely quantified *CDKN1A*, *TP53*, and *MDK* transcripts. Both serum availability and line type affected HKG expression. *UBC* was fourfold down-regulated and *HPRT1* 1.75-fold up-regulated in re-fed HT29 cultures. Line-to-line variability in HKG expression was more pronounced than that caused by altering serum availability and could be found even between isogenic cell lines. *PPIA*, *RPLP0*, *YWHAZ*, and *IPO8* were repeatedly highly ranked while *ACTB*, *B2M*, *UBC*, and *PGK1* were ranked poorly. Normalization against *PPIA*/*RPLP0*/*SDHA* was found optimal for studies involving various colon adenocarcinoma cell lines subjected to manipulations of serum availability. We found HKG expression to vary, more pronouncedly by line type than growth conditions with significant differences also between isogenic cell lines. Although using line-specific normalizers remains optimal, a set of pan-line HKG that yields good estimation of relative expression of target genes was proposed.

## Background

Real-time (quantitative) reverse transcription PCR (RT-qPCR) is frequently employed for unravelling the pathomechanisms of diseases to aid the research on new potential biomarkers and therapeutic strategies (Bustin and Murphy 2013). Normalization against unregulated genes, called “housekeeping” genes (HKG), is a common way to account for a non-biological variation introduced during sample handling and thus to avoid quantification errors. However, a body of evidence has gathered showing that HKG expression may in fact vary between different tissues or cell lines and change in response to pathology, treatment, or altered environmental conditions (Dheda et al. 2005). Moreover, glyceraldehade-3-phosphate dehydrogenase (GAPDH), the most frequently used normalizer, has been demonstrated to increase over 40-fold in severe sepsis (Cummings et al. 2014) but decrease with ageing (Vigelsø et al. 2015). Concerning cancer, GAPDH confers growth advantage and hence is frequently up-regulated in tumor cells (reviewed in Guo et al. 2013; Ramos et al. 2015). Alterations in HKG expression may be too subtle to affect the results obtained by semi-quantitive methods like end-point PCR or to manifest themselves at protein level. However, standardization against inappropriate HKG may lead to invalid conclusions when much more sensitive assays like quantitative real-time PCR are used as shown by Caradec et al. (2010) demonstrating a false *PAR1* up-regulation in LNPCaP cells grown in response to hypoxia following normalization against unstable HKG. Therefore, a necessity of HKG validation for various experimental settings, if RT-qPCR is to be used, is increasingly recognized.

Serum withdrawal, with or without subsequent re-supplementation (serum induction), is a frequently used laboratory procedure, whether it is conducted for creating better defined environment for growing cells, to synchronize their growth, or to study mechanisms involved in stress response, apoptosis and autophagy. It may also serve for establishing an experimental model of conditions associated with nutrient-deprivation, e.g. mimic tumor milieu, where faulty blood vessels inefficiently supply cancer cells not only with oxygen but with nutrients as well (Pirkmajer and Chibalin 2011). Although limitation of oxygen availability occurred to have a profound impact on stability of HKG expression (Caradec et al. 2010), data on the possible effect of serum withdrawal and subsequent induction are scanty. Schmittgen and Zakrajsek (2000) reported a several-fold increase in *GAPDH* and *ACTB* expression, but not that of *B2M*, in NIH 3T3 fibroblasts upon serum induction while Pirkmajer and Chibalin (2011) observed GAPDH protein level to be decreased in starving primary human myotubes.

Instability of HKG expression has already been demonstrated for normal and cancerous tissue samples obtained from CRC patients (Sørby et al. 2010; Kheirelseid et al. 2010) as well as normal and inflamed bowel of patients with inflammatory bowel disease (Krzystek-Korpacka et al. 2014). However, the issue has not been systematically addressed in colon adenocarcinoma cell cultures yet. Hence, this study was designed to test the effect of growth conditions and line type on expression of fifteen commonly used HKG in order to find relatively stable normalizers to be used in in vitro experiments on colon adenocarcinoma cell cultures involving serum-withdrawal and induction. HKG suitability was verified by comparing the expression of *CDKN1A* (p21CIP1/WAF1), *TP53* (tumor protein p53), and *MDK* (midkine) calculated using both absolute and relative quantification methods. We found HKG expression to vary, more pronouncedly by line type than growth conditions with significant differences in the expression of some HKG also between isogenic cell lines. Relatively stable line-specific and pan-line HKG were identified. The impact of using inappropriate reference genes ranging from affecting statistical outcome to drawing false conclusions was demonstrated.

## Materials and methods

### Cell cultures

Seven authenticated human colon adenocarcinoma cell lines (ATCC) were obtained from the Polish Collection of Microorganisms (PCM) of the Institute of Immunology and Experimental Therapy of Polish Academy of Science, Wroclaw, Poland: Lovo (PCM-TC080 = ATCC: CCL-229), HT29 (PCM-TC044 = ATCC: HTB-38), SW620 (PCM-TC046 = ATCC: CCL-227), SW480 (PCM-TC160 = ATCC: CCL-227), HCT116 (PCM-TC161 = ATCC: CCL-247), Caco-2 (PCM-TC017 = ATCC: HTB-37), and DLD-1 (PCM-TC162 = ATCC: CCL-221). Cells were grown on 75 cm^2^ cell culture flasks (BD Bioscience, San Jose, CA, USA) in DMEM/F12 medium (Life Technologies, Carlsbad, CA, USA), supplemented with 10 % FBS (v/v) and 1 % (v/v) l-glutamine-penicillin–streptomycin until 80 % confluence, then harvested using TrypLE Express (Life Technologies), and counted with Countess(R) Automated Cell Counter (Life Technologies). Subsequently, 1 × 10^6^ cells/well were seeded on plastic 6-well flat bottom culture plates (BD Bioscience), cultured for 24 h at 37 °C in a humidified atmosphere containing 5 % CO_2_. The complete medium was then replaced with serum-free medium for 24 h and, subsequently, half of the cells received a new portion of serum-free medium and the other half was re-fed by replacing serum-free medium with a complete one (supplemented with 10 % FBS). Cells were harvested at two time points: after 24 and 48 h following media replacement. Upon termination, supernatants were removed and cells were scratched and lysed with 1 ml of TRI Reagent (Sigma-Aldrich, St. Louis, MO, USA) and stored at −80 °C until RNA isolation. For each cell line, two complete sets of cells cultured in parallel for 24 and 48 h, under both 0 and 10 % FBS were available.

### RNA extraction, quantitation and quality assessment

Cell lysates were centrifuged upon refreezing (12,000×*g*, 4 °C, 10 min) and chloroform was added to the supernatant (0.2 ml per 1 ml of TRI Reagent), mixed, and centrifuged after 5 min incubation at RT (12,000×*g*, 4 °C, 15 min). RNA-containing aqueous upper phase was collected and passed through gDNA Eliminator spin columns and then purified using RNeasy Plus Mini Kit (Qiagen, Hilden, Germany) according to manufacturer’s instructions. Isolated RNA was quantified by means of UV spectroscopy with NanoDrop 2000 (Thermo Scientific, Rockford, IL, USA), measured in duplicates, and its purity assessed by calculating ratios of absorbances at 260, 280, and 230 nm. RNA integrity was assessed using the Experion automated electrophoresis platform incorporating LabChip microfluidic technology and Experion RNA StdSens analysis kits (BioRad, Hercules, CA, USA). The RNA quality indicator (RQI) grading RNA from 10 (intact RNA) to 1 (degraded RNA) was calculated by Experion software for all samples. Possible presence of inhibitors in each RNA isolate was tested by calculating RT-qPCR reaction efficiencies from standard curves prepared by serial dilutions of respective cDNA samples (fivefold dilutions, 6 point-curve, conducted in duplicates).

### cDNA synthesis

1 µg of purified RNA from cell culture samples per reaction (20 µl) was reversely transcribed using Maxima First Strand cDNA Synthesis Kit for RT-qPCR (Thermo Scientific), containing modified M-MuLV reverse transcriptase, RiboLock™ RNase inhibitor, and a mixture of oligo (dT)_18_ and random hexamer primers, according to the manufacturer’s protocol: 10 min incubation at 25 °C, 30 min incubation at 50 °C, and reaction termination by heating samples at 85 °C for 5 min, all in C1000 termocycler (BioRad). Negative transcription (no-RT) controls, devoid of reverse transcriptase, were prepared for all samples.

### RT-qPCR

We evaluated the following HKG: *ACTB*, *B2M*, *GAPDH*, *GUSB*, *HPRT1*, *IPO8*, *MRPL19*, *PGK1*, *PPIA*, *RPLP0*, *RPS23*, *SDHA*, *TBP*, *UBC*, and *YWHAZ*. Full gene names, accession numbers as well as functions of encoded proteins and the sequences of specific, intron-spanning primers (designed and tested for specificity as previously described (manuscript submitted)) are listed in Table 1. Primers’ efficiencies (Table 1) were determined with RT-qPCR and a mixture of DNA templates used in this experiment.

**Table 1:** Sequences and efficiency of primers used in current study

Symbol	Gene name and function of encoded protein	Accession no.	Primer sequence 5′ → 3′ (forward/reverse)	Amp. size (bp)	E (%)
*ACTB* ^a^	Actin, β; structural protein cytoskeleton	NM_001101.3	F: caccattggcaatgagcggtt R: aggtctttgcggatgtccacgt	135	104.2
*B2M* ^a^	β-2-microglobulin; β-chain of MHC class I molecules	NM_004048.2	F: ccactgaaaaagatgagtatgcct R: ccaatccaaatgcggcatcttca	126	95.7
*GAPDH* ^a^	Glyceraldehyde-3-phosphate dehydrogenase; enzyme of glycolytic pathway	NM_002046.4	F: gtctcctctgacttcaacagcg R: accaccctgttgctgtagccaa	131	105.8
*GUSB*	β-Glucuronidase, lysosomal exoglycosidase	NM_000181	F: ctgtacacgacacccaccac R: attcgccacgactttgtt	159	92.6
*HPRT1*	Hypoxanthine phosphoribosyl-transferase; purine metabolism	NM_000194.2	F: tgacactggcaaaacaatgca R: ggtccttttcaccagcaagct	94	105.1
*IPO8*	Importin 8; nuclear protein import	NM_006390.3	F: tggtatggtggaagtgtaagaagtg R: ttggttgagatagttgaatgcttgc	230	107.1
*MRPL19* ^a^	Mitochondrial ribosomal protein L19	NM_014763.3	F: caggaagaggacttggagctac R: gctatcatccagccgtttctcta	137	93.8
*PGK1* ^a^	Phosphoglycerate kinase 1; glycolytic enzyme	NM_000291.1	F: ccgctttcatgtggaggaagaag R: ctctgtgagcagtgccaaaagc	149	107.1
*PPIA* ^a^	Peptidylprolyl isomerase A; protein folding	NM_021130.3	F: ggcaaatgctggacccaacaca R: tgctggtcttgccattcctgga	161	104.6
*RPLP0* ^a^	Ribosomal protein, large, P0; component of 60S subunit	NM_001002.3	F: tggtcatccagcaggtgttcga R: acagacactggcaacattgcgg	119	106.4
*RPS23* ^a^	Ribosomal protein S23; component of 40S subunit	NM_001025.4	F: aggaagtgtgtaagggtccagc R: caccaacagcatgacctttgcg	142	106.9
*SDHA*	Succinate dehydrogenase subunit A; subunit of respiratory chain complex	NM_004168.2	F: agaggcacggaaggagtcac R: caccacatcttgtctcatcagtagg	267	95.9
*TBP*	TATA-box-binding protein; general transcription factor	NM_003194.4	F: tataatcccaagcggtttgctg R: ctggctcataactactaaattgttg	283	102.2
*UBC*	Ubiquitin C; protein degradation	NM_021009.5	F: ggaacaggcgaggaaaagtagtc R: gtcttaccagtcagagtcttcacg	209	96
*YWHAZ*	Tyrosine 3-monooxygenase/tryptophan 5-monooxygenase activation protein, zeta polypeptide; signal transduction	NM_003406.3	F: tcacaacaagcataccaagaagc R: gtatccgatgtccacaatgtcaag	263	97.4

Remaining primers were designed using Beacon Designer Probe/Primer Design Software (BioRad) as previously described (manuscript submitted)

Forward and reverse primer sequences are denoted by “F” and “R”, respectively

*Amp.* amplicon, *E* efficiency

^a^ primer sequences were as proposed by Origene (www.origene.com)

Samples were assessed in three technical replicates (within the same run) and accompanied by respective no-RT controls as well as no template control. To minimize inter-run variation, the same gene was tested in the same analytical run on different samples; each cDNA was diluted from stock once, aliquoted, and stored at −80 °C; all genes were tested on a series of samples within 2–3 days to avoid prolonged storage of diluted cDNA.

All RT-qPCR reactions were conducted with CFX96 Real-Time PCR system (BioRad) using SsoFast EvaGreen^®^ Supermix (BioRad), containing Sso7d-fusion polymerase and EvaGreen dye and the following cycling conditions: 30 s activation at 95 °C, 5 s denaturation at 95 °C, annealing/extension for 5 s at 61 °C, 40 cycles, followed by melting step (60–95 °C with fluorescent reading every 0.5 °C). Reaction mixture contained 2 µl of diluted 1:10 cDNA, 10 µl of 2 × SsoFast EvaGreen^®^ Supermix, 1 µl of each 10 nM forward and reverse target-specific primers, and water up to 20 µl.

Additionally, the absolute quantification of three target genes: *CDKN1A* (encoding p21^CIP1/WAF1^ protein), *TP53* (encoding tumor protein p53), and *MDK* (encoding midkine, a pro-tumorigenic cytokine) was conducted for comparative purposes. For this, standard curves based on serial tenfold dilutions of *CDKN1A*, *TP53*, or *MDK* transcripts cloned into pJET1.2 plasmid (10^9^ to one copy per ml) (ThermoScientific) were prepared. Mean plasmid DNA concentrations measured with NanoDrop 2000 were 20.6, 26.9, and 11.23 ng/µl, respectively.

### Statistical analysis

Technical replicates were averaged prior to any analyses. Expression stability was evaluated using two different statistical approaches, namely by calculating (1) intra- and inter-group variability combined into stability value, derived using NormFinder software version 0.953 (available as MS Excel Add-in at www.mdl.dk.publicationsnormfinder.htm) (Andersen et al. 2004), and (2) the average pairwise variation of a specific gene as compared with other genes, derived using geNorm utility in qbasePLUS version 2.4 software (Biogazelle BE, Ghent, Belgium) (Vandesompele et al. 2002). NormFinder generates a stability value for each gene, which is a direct measure for the estimated expression variation. It allows ranking genes according to the similarity of their expression profiles with lower values indicative of higher stability. Similarly, GeNorm generates M value for each gene with a lower value representative of increased gene stability across samples. GeNorm M value below 1.5 is arbitrarily suggested to be acceptable expression stability. GeNorm generates also V value, which is a pairwise stability measure to determine the benefit of adding extra reference genes for the normalization process with 0.15 as an arbitrary cut-off.

Data were uploaded as suggested by software designers: in an efficiency-corrected linearized form using the following expression: Eamp^-Cq, where Eamp = 10^(1/-slope of target standard curve) for NormFinder and as efficiency corrected Cq values for geNorm. Relative expression of target genes (*CDKN1A*, *TP53*, and *MDK*) was calculated using qbasePLUS.

The effect of growth conditions (serum availability or time) on HKG expression in each cell line was tested on relative quantities, log-transformed if necessary, using paired t-test while the impact of line type with Kruskal–Wallis *H* test. Relative gene expression in isogenic cell lines was compared using unpaired t-test. Data distribution was tested using Kolmogorov–Smirnov test and homogeneity of variances using Levene’s test. All calculated probabilities were two-tailed and *p* values ≤0.05 were considered statistically significant. The analyses were performed using MedCalc Statistical Software version 12.7.5 (MedCalc Software bvba, Ostend, Belgium; http://www.medcalc.org; 2013).

## Results

All RNA isolates obtained from cell cultures were of very good quality with appropriate purity: mean 260/280 absorbance ratio was 2.07 ± 0.03 and mean 260/230 ratio was 2.03 ± 0.39 and high integrity: mean RQI = 9.4 ± 0.87 (range 7.3–10). Mean efficiency derived from dilution series of resulting cDNA templates was 103.6 ± 3.5 % (range 96.4–109.9 %), mean regression coefficient and slope of respective curves was 0.998 ± 0.002 and 3.239 ± 0.077 (range −3.411 to −3.106).

### Effect of serum availability, length of culturing and line type on HKG expression: non-normalized data

**Fig. 1:**
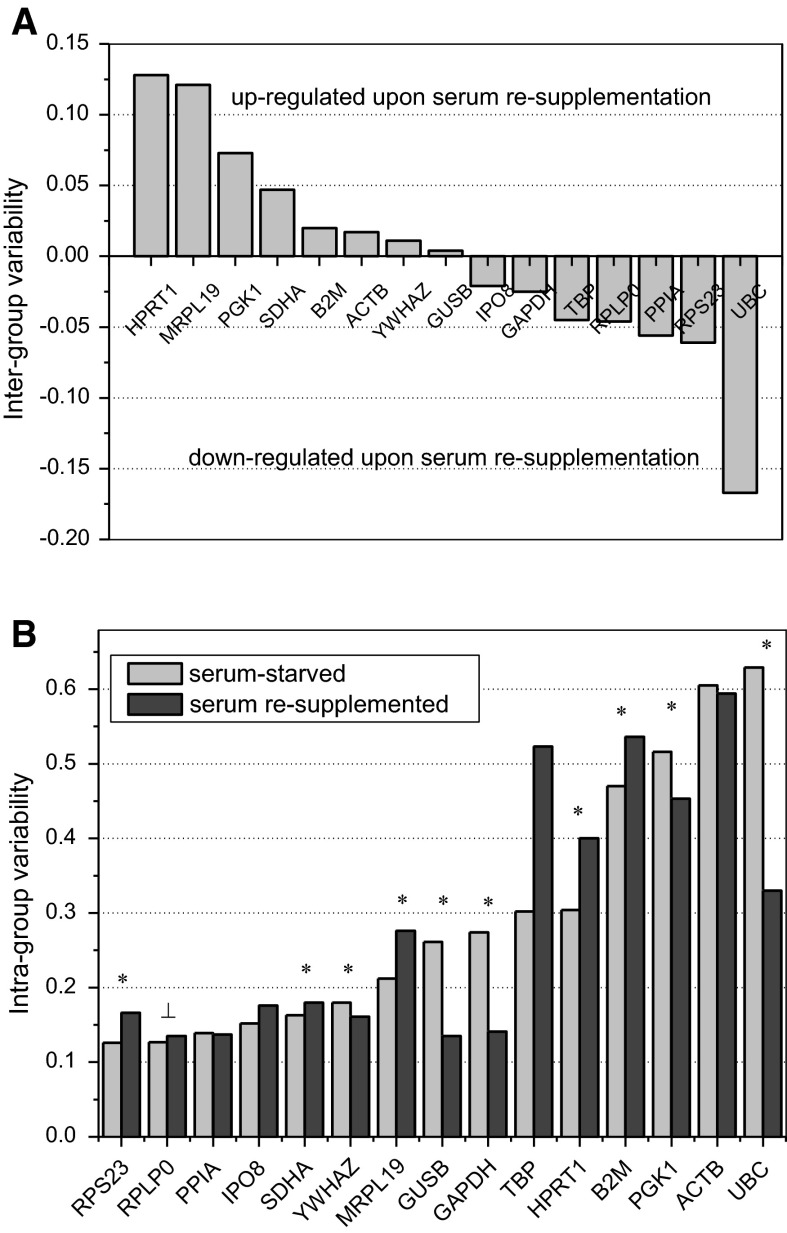
Variability in HKG expression across seven colon adenocarcinoma lines grown with or without serum supplementation. **a** Inter-group variability with groups defined by serum availability. **b** Intra-group variability encompassing the effect of line type, length of culturing, and differences between biological replicates, assessed separately for serum-starved and serum-induced cultures. Bars represents NormFinder estimated inter- and intra-group variability with lower values indicative of more stable expression. Values above Y = 0 show candidate genes that are up-regulated upon serum re-supplementation (down-regulated during prolonged starvation) and values below show HKG that are down-regulated upon serum re-supplementation (up-regulated by prolonged starvation). *Asterisk* statistically significant differences in expression by line type (Kruskal–Wallis *H* test); *statistically significant differences in expression by length of culturing (*t* test for paired samples)

To evaluate the potential effect the growth conditions and line type might have upon HKG expression, we calculated inter- and intra-group variability using NormFinder algorithm. Across all evaluated cell lines, the highest inter-group variability was displayed by UBC (commonly down-regulated upon serum re-supplementation) and by *HPRT1* and *MRPL19* (commonly up-regulated) (Fig. 1a). Subsequently, we compared the relative quantities of these genes in individual cell lines using paired t-test. The analysis showed *UBC* down-regulation to be statistically significant in HT29 cells (*p* = 0.004) and *HPRT1* and *MRPL19* up-regulation statistically significant in, respectively, HT29 (*p* = 0.045) and SW480 (*p* = 0.026) cell lines.

The combined effect of line type, length of culturing, and biological replicates on HKG is depicted in Fig. 1b as an intra-group variability calculated by NormFinder. Overall, its magnitude was higher than for alterations in serum availability. The expression of *UBC*, *ACTB*, *PGK1*, *B2M*, *HPRT1*, and *TBP* varied the most, both when serum-starved and serum re-supplemented cultures were examined. Subsequent statistical analysis of relative quantities using Kruskal-Wallis *H* test showed significant line-to-line differences in the expression of *RPS23* (*p* = 0.008), *B2M* (*p* < 0.001), *GAPDH* (*p* = 0.020), *GUSB* (*p* < 0.001), *HPRT1* (*p* < 0.001), *MRPL19* (*p* = 0.004), *PGK1* (*p* < 0.001), *SDHA* (*p* = 0.027), *UBC* (*p* < 0.001) and *YWHAZ* (*p* = 0.003). While the differences in expression of *RPS23*, *GAPDH*, and *MRPL19* were limited to one or two cell lines (e.g. *GAPDH* expression differed significantly in DLD-1 cells as compared to other lines), the pair-wise comparison for *UBC* or *B2M* yielded number of significant differences.

Interestingly, even the isogenic cell lines SW480 (primary colon adenocarcinoma) and SW620 (its lymph node metastasis) significantly differed by *SDHA* and *GUSB* expression.

All genes were stably expressed overtime except for *RPLP0*, significantly up-regulated in 48 h cultures of HT29 (*p* = 0.035) and Lovo (*p* = 0.032).

Only the variation in the expression of *ACTB*, *TBP*, *IPO8*, and *PPIA*, induced by growth conditions or line type or both, was not statistically significant when non-normalized relative quantities were analyzed.

### Pan-line normalizers

Two popular statistical approaches (NormFinder and geNorm algorithms) were employed to evaluate HKG stability across all cell lines and growth conditions and to select optimal pan-line normalizers. The evaluated genes were ranked from these with the highest stability, indicated by the lowest NormFinder stability value or geNorm M value, to the lowest stability, denoted by the highest scores (Table 2). Although the exact order differed, the same HKG, namely, *RPLP0*, *IPO8*, *GUSB*, *YWHAZ*, and *PPIA*, were highly ranked regardless of the algorithm used and the same genes, namely *ACTB*, *B2M*, *UBC*, and *PGK1*, were found the least stable. *GAPDH*, the most commonly used reference gene, was middle ranked by both algorithms. However, its scores (stability value and M value, respectively) did not differ from the better ranked HKG by much.

**Table 2:** Ranking of HKG expression stability across all cell lines grown under serum-free or serum–supplemented conditions calculated using various statistical approaches (in descending order)

NormFinder stability value*		GeNorm M value^⊥^	
*RPLP0*	0.081	*PPIA*	0.671
*PPIA*	0.084	*RPLP0*	0.674
*IPO8*	0.084	*SDHA*	0.685
*YWHAZ*	0.084	*IPO8*	0.7
*RPS23*	0.086	*GUSB*	0.714
*GUSB*	0.088	*RPS23*	0.725
*SDHA*	0.089	*YWHAZ*	0.767
*GAPDH*	0.092	*GAPDH*	0.801
*MRPL19*	0.108	*MRPL19*	0.84
*HPRT1*	0.124	*TBP*	0.873
*TBP*	0.126	*HPRT1*	0.913
*B2M*	0.138	*PGK1*	0.966
*PGK1*	0.138	*B2M*	1.009
*UBC*	0.141	*UBC*	1.042
*ACTB*	0.150	*ACTB*	1.08
*RPLP0* and *SDHA*	0.056	*PPIA, RPLP0* and *SDHA* ^#^	

Data presented as stability values calculated for each HKG using NormFinder or GeNorm algorithms. A set of genes, the combination of which provides increased stability is presented in the last row (stability value of a set is calculated exclusively by NormFinder)

* Norm Finder stability value is a direct measure for the estimated expression variation. Lower values are indicative of higher expression stability

^⊥^ GeNorm M value indicates gene expression stability across samples with lower values representing increased stability. Arbitrarily, M values <1.5 are indicative of acceptable expression stability

^#^ The improvement of the GeNorm value is not shown for the combination of *PPIA*, *RPLP0*, and *SDHA*

NormFinder found *RPLP0* the most stably expressed single HKG, followed by *PPIA* and *IPO8*. However, the software suggested *RPLP0* and *SDHA*, the fourth HKG in rank, as an optimal pair of normalizers. As shown by inter-group variability (Fig. 1a), *SDHA* expression in the present sample set is rather up-regulated upon serum re-supplementation what would compensate *RPLP0* down-regulation while the expressions of *PPIA* and *IPO8* tend to be down-regulated as well.

**Fig. 2:**
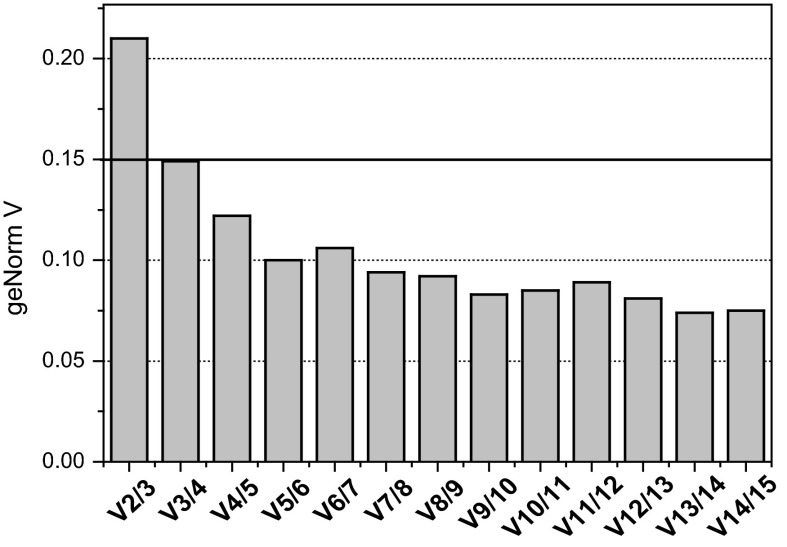
Determination of optimal number of HKG to be used as reference as pan-line normalizers. Optimal number was determined using GeNorm algorithm based on pairwise variation analysis. GeNorm V values represent the benefit of adding extra gene to the set of normalizers, e.g. V2/3 is a comparison of normalization based on two vs. three HKG; V3/4 is a comparison of normalization based on three vs. four HKG, etc. An arbitrary cut off value of 0.15 is indicative of a significant effect and point at the necessity to include the added HKG in a panel of normalizers

According to GeNorm, under study conditions, the average stability of evaluated HKG was medium with average M value >0.5 but ≤1. Optimal number of genes to be used as normalizers in the studied set of samples was calculated to be three, namely *PPIA*, *RPLP0*, and *SDHA*. As depicted on Fig. 2, there was significant improvement in normalization based on three than two HKG (GeNorm V2/3 value exceeded arbitrary cut-off of 0.15). In turn, the effect of introducing the fourth gene was insubstantial (GeNorm V3/4 was <0.15).

### Line-specific normalizers

Using the same approach, we devised line-specific normalizers as well. The resulting geNorm and NormFinder ranking lists were concordant with only small shifts in the positions of specific genes. *UBC/PPIA*, *YWHAZ/RPS23*, *YWHAZ/B2M*, and *GAPDH/PPIA* pairs were found optimal normalizers, respectively for DLD-1, SW480, HCT116, and Caco-2 lines by NormFinder (Table 3), while *GUSB/RPLP0*, *RPS23/RPLP0*, *UBC/RPLP0/B2M*, and *GUSB/YWHAZ* by geNorm (Table 4). For HT29, SW620, and Lovo both approaches yielded the same pairs of HKG, respectively, *YWHAZ/B2M*, *YWHAZ/IPO8*, and *GUSB/YWHAZ*.

**Table 3:** Line-specific HKG expression stability in cell lines grown under serum-free or serum–supplemented conditions ranked by increasing stability value calculated with NormFinder software

DLD-1		HT29		SW480		SW620		HCT116		Caco-2		Lovo	
*UBC*	.047	*YWHAZ*	.115	*SDHA*	.157	*YWHAZ*	.062	*B2M*	.110	*PPIA*	.081	*YWHAZ*	.046
*PPIA*	.061	*B2M*	.123	*GUSB*	.174	*IPO8*	.097	*YWHAZ*	.136	*GAPDH*	.108	*GUSB*	.050
*RPLP0*	.076	*TBP*	.142	*YWHAZ*	.195	*B2M*	.140	*PGK1*	.145	*GUSB*	.114	*B2M*	.065
*RPS23*	.078	*RPLP0*	.169	*RPS23*	.198	*GUSB*	.157	*RPLP0*	.147	*B2M*	.131	*RPS23*	.076
*SDHA*	.085	*SDHA*	.221	*B2M*	.200	*RPLP0*	.158	*UBC*	.168	*IPO8*	.137	*HPRT1*	.096
*HPRT1*	.093	*IPO8*	.236	*RPLP0*	.210	*HPRT1*	.163	*RPS23*	.186	*RPS23*	.140	*PPIA*	.117
*YWHAZ*	.094	*GAPDH*	.258	*GAPDH*	.226	*GAPDH*	.169	*PPIA*	.202	*PGK1*	.153	*UBC*	.169
*GUSB*	.109	*RPS23*	.270	*IPO8*	.256	*RPS23*	.208	*GUSB*	.229	*MRPL19*	.156	*ACTB*	.172
*IPO8*	.123	*PPIA*	.274	*PPIA*	.256	*PPIA*	.210	*HPRT1*	.252	*TBP*	.164	*RPLP0*	.197
*MRPL19*	.139	*GUSB*	.277	*HPRT1*	.268	*SDHA*	.212	*SDHA*	.262	*RPLP0*	.188	*MRPL19*	.198
*GAPDH*	.149	*PGK1*	.348	*UBC*	.299	*MRPL19*	.283	*GAPDH*	.302	*ACTB*	.190	*GAPDH*	.222
*B2M*	.155	*ACTB*	.357	*PGK1*	.312	*UBC*	.304	*MRPL19*	.327	*HPRT1*	.248	*SDHA*	.241
*PGK1*	.215	*HPRT1*	.362	*TBP*	.327	*TBP*	.307	*IPO8*	.333	*SDHA*	.264	*IPO8*	.243
*TBP*	.231	*MRPL19*	.383	*MRPL19*	.349	*ACTB*	.382	*ACTB*	.378	*UBC*	.272	*TBP*	.258
*ACTB*	.356	*UBC*	.661	*ACTB*	.480	*PGK1*	.400	*TBP*	.554	*YWHAZ*	.309	*PGK1*	.531
*PPIA* and *UBC*	.039	*B2M* and *YWHAZ*	.069	*RPS23* and *YWHAZ*	.098	*IPO8* and *YWHAZ*	.059	*B2M* and *PGK1*	.093	*GAPDH* and *PPIA*	.071	*GUSB* and *YWHAZ*	.035

Data presented as stability values calculated for each HKG using NormFinder. A set of genes, the combination of which provides increased stability is presented in the last row. Lower values are indicative of higher expression stability

**Table 4:** Line-specific HKG expression stability in cell lines grown under serum-free or serum–supplemented conditions ranked by increasing GeNorm M value calculated with qbasePLUS software

DLD-1		HT29		SW480		SW620		HCT116		Caco-2		Lovo	
*YWHAZ*	.005	*YWHAZ*	.005	*RPLP0*	.004	*RPS23*	.001	*HPRT1*	.010	*UBC*	.010	*B2M*	.022
*RPLP0*	.005	*GAPDH*	.006	*IPO8*	.005	*SDHA*	.002	*SDHA*	.012	*GUSB*	.011	*GUSB*	.024
*MRPL19*	.007	*TBP*	.007	*RPS23*	.006	*IPO8*	.003	*B2M*	.015	*RPS23*	.013	*SDHA*	.026
*PPIA*	.011	*RPLP0*	.013	*B2M*	.023	*TBP*	.010	*TBP*	.018	*MRPL19*	.032	*PPIA*	.065
*B2M*	.022	*PGK1*	.025	*UBC*	.060	*YWHAZ*	.020	*RPLP0*	.020	*B2M*	.038	*HPRT1*	.087
*HPRT1*	.033	*B2M*	.060	*PPIA*	.075	*GUSB*	.030	*YWHAZ*	.030	*IPO8*	.048	*IPO8*	.100
*RPS23*	.050	*GUSB*	.085	*SDHA*	.130	*GAPDH*	.050	*MRPL19*	.040	*PPIA*	.065	*ACTB*	.115
*GUSB*	.060	*IPO8*	.100	*GAPDH*	.180	*PGK1*	.070	*GAPDH*	.050	*GAPDH*	.080	*MRPL19*	.125
*UBC*	.068	*PPIA*	.110	*GUSB*	.205	*ACTB*	.090	*GUSB*	.062	*YWHAZ*	.105	*YWHAZ*	.140
*IPO8*	.080	*RPS23*	.125	*YWHAZ*	.230	*PPIA*	.108	*ACTB*	.070	*SDHA*	.120	*RPS23*	.170
*PGK1*	.098	*SDHA*	.137	*HPRT1*	.262	*UBC*	.123	*UBC*	.080	*RPLP0*	.132	*RPLP0*	.190
*SDHA*	.110	*ACTB*	.155	*PGK1*	.285	*RPLP0*	.142	*IPO8*	.122	*HPRT1*	.142	*GAPDH*	.210
*GAPDH*	.130	*MRPL19*	.175	*TBP*	.337	*MRPL19*	.160	*PPIA*	.170	*TBP*	.168	*UBC*	.240
*TBP*	.142	*HPRT1*	.225	*ACTB*	.410	*B2M*	.218	*RPS23*	.212	*ACTB*	.212	*TBP*	.270
*ACTB*	.165	*UBC*	.405	*MRPL19*	.460	*HPRT1*	.258	*PGK1*	.262	*PGK1*	.243	*PGK1*	.315
*RPLP0* and *YWHAZ*		*GAPDH* and *YWHAZ*		*IPO8* and *RPLP0*		*SDHA* and *RPS23*		*SDHA* and *HPRT1*		*GUSB* and *UBC*		*GUSB* and *B2M*	

Data are presented as stability values (M) calculated for each HKG using GeNorm algorithm. A set of genes, the combination of which provides increased stability is presented in the last row (GeNorm does not provide M value for combination of selected genes). Lower values are indicative of increased stability

However, some striking differences in gene stability were found between lines. Regardless the algorithm used, *UBC* was top-ranked in DLD-1 cells but worst-ranked in HT29. Similarly, stability of *YWHAZ* was highly ranked in all cell lines except for Caco-2. *RPLP0* was generally well-rated except for Caco-2 and Lovo lines, while *PGK1* was generally ranked poorly except for HCT116. *TBP* was one of top-ranked HKG in HT29 but otherwise ranked poorly and *IPO8* occupied high positions on SW620 list but last ones on HCT116 list.

**Fig. 3:**
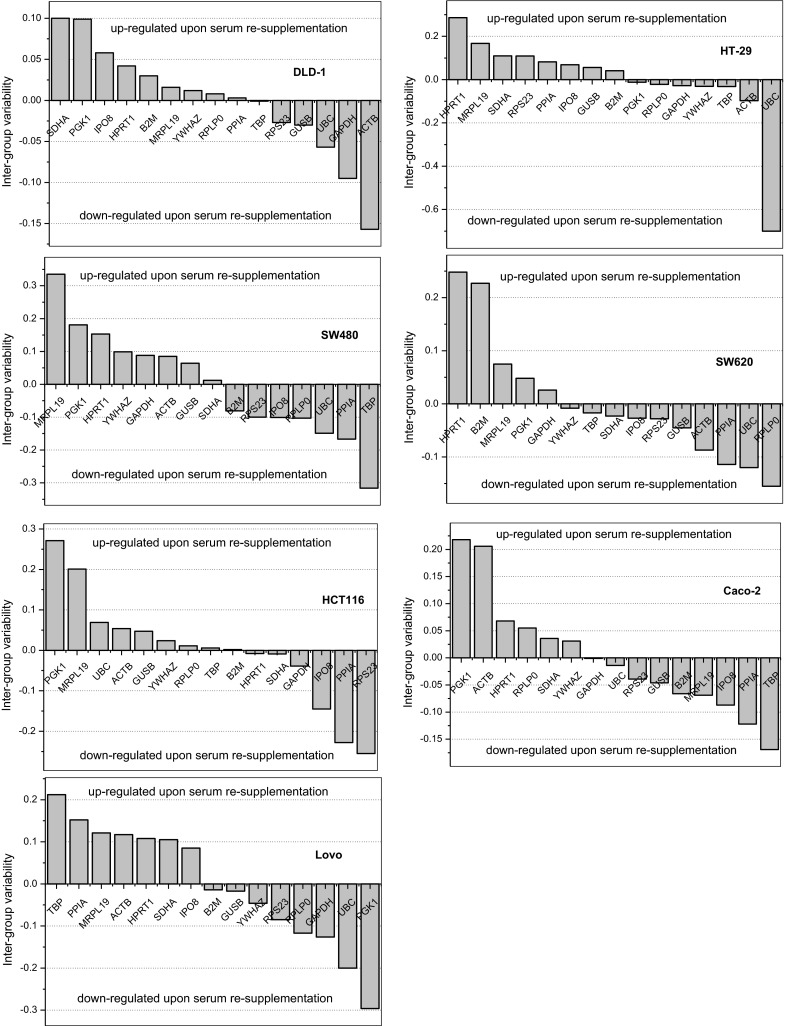
Inter-group variability in HKG expression in individual cell lines. Bars represent NormFinder estimated inter-group variability with groups defined by serum availability. Lower values are indicative of more stable expression. Values above Y = 0 show candidate genes that are up-regulated upon serum re-supplementation (down-regulated during prolonged starvation) and values below show HKG that are down-regulated upon serum re-supplementation (up-regulated by prolonged starvation)

As shown in Fig. 3, there were line-to-line differences in their response to serum induction as well, e.g. *ACTB* was up-regulated in Caco-2 cells and Lovo but down-regulated in DLD-1 while *PGK1* was up-regulated in Caco-2 but down-regulated in Lovo. Also the isogenic cell lines differ: *B2M* was rather down-regulated upon serum re-supplementation in SW480 but up-regulated in SW620, *ACTB* was rather up-regulated in SW480 but down-regulated in SW620, and *TBP* was down-regulated in SW480 but its expression was not affected in SW620.

### Validation of devised normalizers

In order to validate the devised sets of HKG, we compared relative expression ratios (normalized expressions in serum-induced to serum-starved cultures) obtained using various combinations of reference genes with the one resulting from absolute quantification with a copy number. HKG performance was tested on three target genes, the expression of which was evaluated in 48 h cultures of HT29, Caco-2, and DLD-1 cells. Apart from pan-line normalizers devised by geNorm or NormFinder, we constructed another set consisting of HKG that were not significantly affected by line type or culture growth conditions, that is, *ACTB*, *TBP*, *IPO8*, and *PPIA*.

**Fig. 4:**
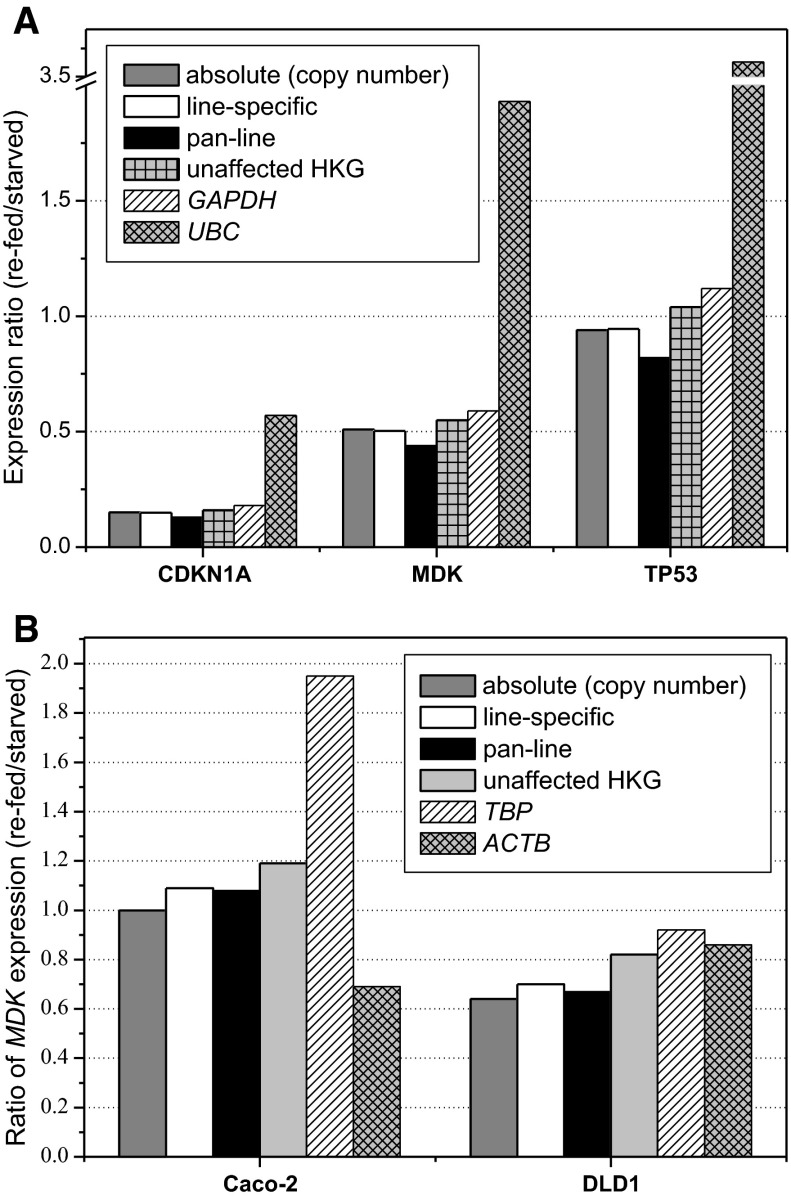
Comparison of absolute and relative quantification using various normalizers. **a** Relative expression of genes of interest, *CDKN1A*, *MDK*, and *TP53*, in 48 h cultures of HT29 evaluated using number of template copies (absolute quantification) or normalized using: line-specific set of HKG (*YWHAZ*/*B2M*), pan-line set of HKG (*RPLP0*/*SDHA*/*PPIA*), set of candidate HKG found unaffected significantly by any variable in the study (*ACTB*/*TBP*/*PPIA*/*IPO8*), *GAPDH* as the commonest arbitrarily chosen HKG, and *UBC* as the least stable reference gene in HT29 cell line but the most stable in others. **b** Relative expression of *MDK* in 48 h cultures of Caco-2 and DLD-1 cells evaluated using number of template copies (absolute quantification) or normalized using: line-specific set of HKG (*RPS23*/*B2M* and *GUSB*/*RPLP0*), pan-line set of HKG (*RPLP0*/*SDHA*/*PPIA*), set of “unaffected” HKG (*ACTB*/*TBP*/*PPIA*/*IPO8*), *TBP* and *ACTB* as genes characterized by high variability in Caco-2 (oppositely directed and hence compensating) and DLD-1 (no compensation) cell lines. Bars represent the ratio of target gene expression in cultures re-supplemented with serum (serum-fed) to serum-starved

As indicated by 6.7-fold and twofold reduction in DNA copy number, *CDKN1A* and *MDK* expressions were down-regulated upon serum re-supplementation in HT29 cells, while that of *TP53* remained unaffected (Fig. 4a). The same conclusions could be reached whether software-devised line-specific (*YWHAZ/B2M*) or pan-line (*RPLP0/PPIA/SDHA*) normalizers were used. Since the overall *GAPDH* rating (both line-specific and pan-line) was not bad, normalization against this single, commonly used reference gene did not substantially altered study conclusions on target gene expression. However, normalization against the unstable *UBC* underestimated *CDKN1A* down-regulation and led to erroneous conclusions on *MDK* and *TP53* up-regulation in response to serum induction.

Despite uniformly poor ratings of *ACTB* and mediocre/poor of *TBP*, a set of “unaffected” HKG (*ACTB/TBP/IPO8/PPIA*) gave an estimation of changes in target gene expression close to the absolute one (Fig. 4a) Similarly, relating *MDK* expression in Caco-2 cells to *ACTB/TBP/IPO8/PPIA* (Fig. 4b) did not alter experiment conclusion on lack of *MDK* regulation upon serum re-supplementation in this particular cell line. It might be explained by relatively low inter-group variability in *ACTB* and *TBP* expression in HT29 as compared to other lines (Fig. 3, HT29). In Caco-2 cells, in turn, their variability was high but of similar magnitude and oppositely directed, with *ACTB* substantially up- while *TBP* down-regulated (Fig. 3, Caco-2). Hence, the effect of one gene was countered by the other. If *ACTB* or *TBP* were used as sole normalizers, *MDK* would be falsely interpreted as, respectively, down- or up-regulated upon serum induction (Fig. 4b). In DLD-1 cells, *ACTB* displayed substantial variability that was not countered by *TBP* (Fig. 3, DLD-1). In such a case, as demonstrated by *MDK* expression significantly down-regulated by serum re-supplementation (Fig. 4b), software-devised pan-line normalizers were superior. They did not alter experiment conclusion, even though they included genes found significantly affected by growth conditions (*RPLP0*) or line type (*SDHA*). On the contrary, normalizing against a set consisting of “unaffected” but poorly ranked genes underestimated the effect so the statistical significance of *MDK* down-regulation was lost.

### Effect of growth condition and line type on HKG expression: validation on normalized data

**Fig. 5:**
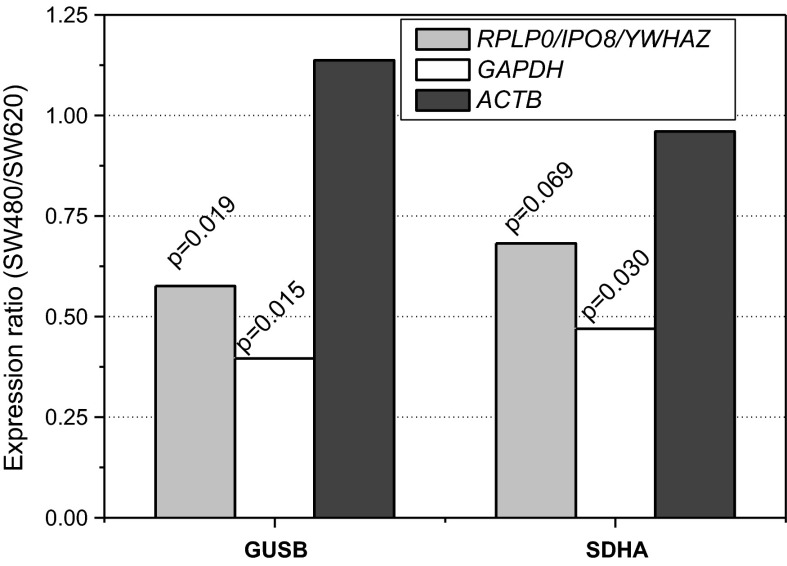
Differences in HKG expression between isogenic cell lines. HKG expression in cell lines derived from the same patient (isogenic cell lines)—SW480 (primary) and SW620 (lymph node metastasis)—were evaluated. *Bars* represent relative expression of *GUSB* and *SDHA* in SW480 to SW620 when normalized against genes found stably expressed in both lines (selected by geNorm: *RPLP0/IPO8/YWHAZ*) and arbitrarily chosen, the commonest reference genes: *GAPDH* and *ACTB*

Statistical analysis on relative quantities (non-normalized) shown line-to-line differences in expression levels of most of the evaluated HKG except for *ACTB*, *TBP*, *RPLP0*, *PPIA*, and *IPO8* to be significant. However, when data were normalized against pan-line normalizers (*RPLP0/SDHA/PPIA*) to account for non-biological variation (e.g. differences in template load or reaction efficiency), pair-wise comparisons of *GAPDH*, *PGK1*, or *RPS23* expression did not yield significant differences. The expression of other genes, previously found affected by line type, remained different. Also two isogenic cell lines, SW480 and SW620, significantly differed by their non-normalized *GUSB* and *SDHA* expression. To verify this finding, we compared their relative expression normalized against geometric mean of *RPLP0*, *IPO8*, and *YWHAZ*, found optimal by geNorm for SW480 and SW620. Relative *GUSB* and *SDHA* expression was up-regulated in SW620 (line derived from secondary tumor), significantly in case of *GUSB* (Fig. 5).

To further demonstrate the importance of using validated normalizers, we estimated relative expression of *GUSB* and *SDHA* using *GAPDH* (middle-rated) or *ACTB* (the worst-ranked) as sole normalizers. While normalization against *GAPDH* would overestimate the difference in expression, using *ACTB* as reference would not show any differences in *GUSB* or *SDHA* expression between lines.

Using normalized data with line-specific reference genes we also verified the findings on HKG expression being affected by length of culturing and serum availability. The difference in *RPLP0* expression between 24 and 48 h cultures of HT29 and Lovo become insignificant (*p* = 0.080 and *p* = 0.426) when normalized against *YWHAZ/B2M* and *YWHAZ/GUSB,* respectively. However, the expression of *UBC* in HT29 cells upon serum induction remained over fourfold down-regulated (*p* = 0.011) following normalization and that of *HPRT1*—1.75-fold up-regulated (*p* = 0.032). Yet, the twofold increase in *MRPL19* transcripts in serum re-supplemented SW480 cells lost significance, whether normalization was based on geNorm (*RPLP0/RPS23*) or NormFinder (*YWHAZ/RPS23*) selected pairs of line-specific HKG (*p* = 0.145 and *p* = 0.259, respectively).

## Discussion

There is a growing awareness that the expression of housekeeping genes, previously believed to be stable, may be affected by experimental settings and that normalization against a single, arbitrary chosen HKG may jeopardize the relevance of a study. Thus, it is suggested that quantitative PCR experiments should be preceded by a thorough examination of expression stability of potential HKG under dedicated conditions (Caradec et al. 2010). Manipulating the availability of serum for varying time periods is a common laboratory practice in molecular biology that may serve purposes as different as preparing cells for the proper experiment by increasing homogeneity of culture and uniformity of growing conditions or constitute an experiment *per se* (Pirkmajer and Chibalin 2011). Although the limited accessibility of nutrients, growth factors, and hormones may potentially affect expression of HKG in a way similar to oxygen deprivation (Caradec et al. 2010), the published data are limited and restricted to fibroblasts and primary cells, entirely depending on serum as a growth factor source (Iyer et al. 1999; Shi et al. 2012), while their potential effect on cancer cell lines is unknown. Schmittgen and Zakrajsek (2000) demonstrated that cultured murine fibroblasts grown for 24 h in serum-free medium and subsequently induced with 15 % FBS increased the expression of *GAPDH* and *ACTB* several-fold, rendering these genes inappropriate as internal controls for studies involving serum withdrawal and induction. Correspondingly, primary human and rat myotubes as well as human embryonic kidney (HEK)293 cells displayed gradually decreasing GAPDH protein content during 24 h serum withdrawal (Pirkmajer and Chibalin 2011). On the other hand, the excess of glucose in culture media (Liu et al. 2016; Bakhashab et al. 2014) or cell stimulation with growth factors (Tratwal et al. 2014) has been demonstrated to affect HKG stability as well.

GAPDH has been outperformed by other HKG also when normal and cancerous tissues were compared (de Kok et al. 2005; Blanquicett et al. 2002; Dydensborg et al. 2006). However, colon adenocarcinoma cell lines, as demonstrated here by rather low intergroup variability both when assessed combined and individually, do not respond to alterations in serum availability by substantial changes in *GAPDH* levels. Hence, normalizing against this HKG did not affect the conclusion of our experiments. Yet, with stability of its expression being suboptimal, it could affect the statistical outcome. Of note, preservation of *GAPDH* expression upon altered conditions has been reported for human umbical vein endothelial cells (HUVECs) grown under hyperglycemic conditions (Bakhashab et al. 2014), chondrocytes cultured at different temperatures (Ito et al. 2014) or blood cells subjected to radiation (Vaiphei et al. 2015).

Concerning *ACTB*, its overall intergroup variability was in the current study low but the expression in particular cell lines was affected by alterations in serum availability, discouraging its application in in vitro studies involving serum withdrawal and re-supplementation. However, since the alterations occurred in both directions, *ACTB* displayed low variability as a pan-line normalizer. Our finding corroborates the observations of other authors on *ACTB* expression varying considerably with changing experimental conditions or between individuals (Caradec et al. 2010; Kheirelseid et al. 2010; Andersen et al. 2004). On the contrary, *ACTB* has been found among the most stably expressed HKG in breast cancer cell lines (Liu et al. 2015). We observed that particularly the expression of *UBC* and *HPRT1* in HT29 and *MRPL19* in SW480 was significantly altered by changes in serum availability disqualifying them as reference genes, even though *UBC* (Andersen et al. 2004) and *HPRT1* (Sørby et al. 2010) were recommended as suitable normalizers for RT-qPCR studies on tissue specimens from CRC patients.

Caradec et al. (2010) demonstrated on prostate carcinoma cells that great expression variability can be found between cell lines derived from the same tissue. As such, the results obtained for one line should not be easily adopted for the other. Accordingly, we found that the observed fluctuations in HKG expression related to serum availability were surpassed by line-to-line differences in gene stability. Substantiating the notion, we found *PGK1* expression to be unaffected by alterations in serum availability in HT29 line. Correspondingly, *PGK1* expression was the most stable one after HT29 challenge with probiotic and pathogenic bacteria as reported by Jacobsen et al. (2014). However, concurrently, we found *PGK1* to be among the most often up- or down-regulated HKG by serum re-supplementation in other colonic epithelial cell lines. The expression of most of the HKG differed significantly between particular cell lines both when non-normalized data were examined and when a non-biological variation was accounted for. *UBC* is a striking example how mechanical extrapolation of results obtained for one line to the other can affect conclusions of the experiment—in our study underestimating the magnitude of *CDKN1A* down-regulation or demonstrating false up-regulation of *MDK* (down-regulated) and *TP53* (unaltered) upon serum re-supplementation in 48 h HT-29 cultures.

Interestingly, the stability of HKG can very also between isogenic cell lines (derived from the same patient), as demonstrated here for primary colonic adenocarcinoma cells (SW480) and their lymph node metastasis (SW620). The expression of *GUSB* and *SDHA* was up-regulated in metastatic cell line as compared to primary one. Also, both lines differ with their response to serum induction with *TBP* expression down-regulated exclusively in primary SW480, *B2M* being up-regulated in metastatic but down-regulated in primary adenocarcinoma, and, oppositely, *ACTB* being down-regulated in metastatic but up-regulated in primary line.

*In vitro* experiments have usually a complex design; still, it is desirable to limit the number of necessary reference genes. Our results revealed that although using line-specific normalizers remains optimal, it is possible to devise a set of reference genes displaying relatively unaltered expression under study conditions. We started expression stability analysis from statistical evaluation of raw data to exclude from investigation genes obviously regulated under experimental conditions and hence unsuited to serve as normalizers. Similarly to other in vitro experiments, there were several variables in our study that might potentially affect HKG expression: line type, length of culturing, and serum availability. As such, the variability in the expression of only four genes was not found significant in response to at least one of the factors. However, this phenomenon, particularly in case of *ACTB* and *TBP*, seems to result from the variability being hard to attribute to any specific factor rather than lack of thereof. As pre-analyses are based on raw data, non-biological variation introduced during sample handling may contribute to observed differences. Accordingly, *RPLP0* was no longer found significantly affected by length of culturing when data were normalized against line-specific normalizers. Consequently, normalization against *ACTB*/*TBP*/*PPIA*/*IPO8* was suboptimal, failing to show significant down-regulation of *MDK* in DLD-1 cells, and was outperformed by HKG set devised by dedicated software from among all genes, without any exclusion.

Regardless algorithm used, *PPIA*, *RPLP0*, and *SDHA* were ranked the most stable in the sample set investigated. Normalization against geometric mean of these HKG yielded results similar to these obtained with line-specific reference genes or with absolute quantification, signifying their reliability as normalizers for RT-qPCR studies on multiple colon adenocarcinoma cell lines involving serum withdrawal and induction. *RPLP0* has been claimed a suitable reference for human intestinal epithelial cells (Dydensborg et al. 2006). In turn, *PPIA* has been repeatedly found a suitable normalizer in a number of human studies (Andrusiewicz et al. 2016; Ali et al. 2015; Lemma et al. 2016), also these concerning CRC patients (Sørby et al. 2010; Kheirelseid et al. 2010), but affected by cell stimulation in others (Kaszubowska et al. 2015). *IPO8* and *GUSB* were yet another HKG recommended for CRC studies (Sørby et al. 2010; Blanquicett et al. 2002) and highly ranked in our in vitro study as well. Analyzing HKG in colon and liver tissues from CRC patients with hepatic metastases, Blanquicett et al. (2002) observed that ribosomal HKG displayed the most stable expression while those involved in metabolic pathways were the least stable ones. Substantiating the notion, we and others (Dydensborg et al. 2006; Bakhashab et al. 2014; Jacobsen et al. 2014) demonstrated superior stability of *RPLP0* and Bian et al. (2015), Powell et al. (2014), and Ito et al. (2014) that of another ribosomal protein—*RPL13A*. In turn, *PGK1* was one of the least stable genes in our study, although *GAPDH*, encoding an enzyme involved in the same metabolic pathway, performed well.

## Conclusions

Expression of commonly used HKG as well as line response to serum withdrawal and induction differ between colon adenocarcinoma cell lines, though these were derived from the same patient (isogenic cell lines). While normalizing against line-specific reference genes is optimal, it is possible to devise common set of HKG, *RPLP0/PPIA/SDHA* in the sample set investigated, suitable for multiline RT-qPCR studies. *GAPDH*, the most popular internal control, occurred to be relatively stably expressed and yet normalizing against it may affect statistical outcome of the study. In turn, using *ACTB*, another frequently used reference, or adopting without validation genes found stable for other lines may lead to invalid conclusions.

## References

[CR1] Ali H, Du Z, Li X, Yang Q, Zhang YC, Wu M, Li Y, Zhang G (2015). Identification of suitable reference genes for gene expression studies using quantitative polymerase chain reaction in lung cancer in vitro. Mol Med Rep.

[CR2] Andersen CL, Jensen JL, Ørntoft TF (2004). Normalization of real-time quantitative reverse transcription-PCR data: a model-based variance estimation approach to identify genes suited for normalization, applied to bladder and colon cancer data sets. Cancer Res.

[CR3] Andrusiewicz M, Słowikowski B, Skibińska I, Wołuń-Cholewa M, Dera-Szymanowska A (2016). Selection of reliable reference genes in eutopic and ectopic endometrium for quantitative expression studies. Biomed Pharmacother.

[CR4] Bakhashab S, Lary S, Ahmed F, Schulten HJ, Bashir A, Ahmed FW, Al-Malki AL, Jamal HS, Gari MA, Weaver JU (2014). Reference genes for expression studies in hypoxia and hyperglycemia models in human umbilical vein endothelial cells. G3 (Bethesda).

[CR5] Bian Z, Yu Y, Quan C, Guan R, Jin Y, Wu J, Xu L, Chen F, Bai J, Sun W, Fu S (2015). RPL13A as a reference gene for normalizing mRNA transcription of ovarian cancer cells with paclitaxel and 10-hydroxycamptothecin treatments. Mol Med Rep.

[CR6] Blanquicett C, Johnson MR, Heslin M, Diasio RB (2002). Housekeeping gene variability in normal and carcinomatous colorectal and liver tissues: applications in pharmacogenomic gene expression studies. Anal Biochem.

[CR7] Bustin SA, Murphy J (2013). RNA biomarkers in colorectal cancer. Methods.

[CR8] Caradec J, Sirab N, Keumeugni C, Moutereau S, Chimingqi M, Matar C, Revaud D, Bah M, Manivet P, Conti M, Loric S (2010). ‘Desperate house genes’: the dramatic example of hypoxia. Br J Cancer.

[CR9] Cummings M, Sarveswaran J, Homer-Vanniasinkam S, Burke D, Orsi NM (2014). Glyceraldehyde-3-phosphate dehydrogenase is an inappropriate housekeeping gene for normalising gene expression in sepsis. Inflammation.

[CR10] de Kok JB, Roelofs RW, Giesendorf BA, Pennings JL, Waas ET, Feuth T, Swinkels DW, Span PN (2005). Normalization of gene expression measurements in tumor tissues: comparison of 13 endogenous control genes. Lab Invest.

[CR11] Dheda K, Huggett JF, Chang JS, Kima LU, Bustin SA, Johnson MA, Rook GAW, Zumla A (2005). The implications of using an inappropriate reference gene for real-time reverse transcription PCR data normalization. Anal Biochem.

[CR12] Dydensborg AB, Herring E, Auclair J, Tremblay E, Beaulieu JF (2006). Normalizing genes for quantitative RT-PCR in differentiating human intestinal epithelial cells and adenocarcinomas of the colon. Am J Physiol Gastrointest Liver Physiol.

[CR13] Guo C, Liu S, Sun MZ (2013). Novel insight into the role of GAPDH playing in tumor. Clin Transl Oncol.

[CR14] Ito A, Aoyama T, Tajino J, Nagai M, Yamaguchi S, Iijima H, Zhang X, Akiyama H, Kuroki H (2014). Evaluation of reference genes for human chondrocytes cultured in several different thermal environments. Int J Hyperthermia.

[CR15] Iyer VR, Eisen MB, Ross DT, Schuler G, Moore T, Lee JCF, Trent JM, Staudt LM, Hudson J, Boguski MS, Lashkari D, Shalon D, Botstein D, Brown PO (1999). The transcriptional program in the response of human fibroblasts to serum. Science.

[CR16] Jacobsen AV, Yemaneab BT, Jass J, Scherbak N (2014). Reference gene selection for qPCR is dependent on cell type rather than treatment in colonic and vaginal human epithelial cell lines. PLoS One.

[CR17] Kaszubowska L, Wierzbicki PM, Karsznia S, Damska M, Ślebioda TJ, Foerster J, Kmieć Z (2015). Optimal reference genes for qPCR in resting and activated human NK cells–Flow cytometric data correspond to qPCR gene expression analysis. J Immunol Methods.

[CR18] Kheirelseid EA, Chang KH, Newell J, Kerin MJ, Miller N (2010). Identification of endogenous control genes for normalisation of real-time quantitative PCR data in colorectal cancer. BMC Mol Biol.

[CR19] Krzystek-Korpacka M, Diakowska D, Bania J, Gamian A (2014). Expression stability of common housekeeping genes is differently affected by bowel inflammation and cancer: implications for finding suitable normalizers for inflammatory bowel disease studies. Inflamm Bowel Dis.

[CR20] Lemma S, Avnet S, Salerno M, Chano T, Baldini N (2016). Identification and validation of housekeeping genes for gene expression analysis of cancer stem cells. PLoS One.

[CR21] Liu LL, Zhao H, Ma TF, Ge F, Chen CS, Zhang YP (2015). Identification of valid reference genes for the normalization of RT-qPCR expression studies in human breast cancer cell lines treated with and without transient transfection. PLoS One.

[CR22] Liu X, Xie J, Liu Z, Gong Q, Tian R, Su G (2016). Identification and validation of reference genes for quantitative RT-PCR analysis of retinal pigment epithelium cells under hypoxia and/or hyperglycemia. Gene.

[CR23] Pirkmajer S, Chibalin AV (2011). Serum starvation: caveat emptor. Am J Physiol Cell Physiol.

[CR24] Powell TR, Powell-Smith G, Haddley K, Mcguffin P, Quinn J, Schalkwyk LC, Farmer AE, D’Souza UM (2014). Mood-stabilizers differentially affect housekeeping gene expression in human cells. Int J Methods Psychiatr Res.

[CR25] Ramos D, Pellín-Carcelén A, Agustí J, Murgui A, Jordá E, Pellín A, Monteagudo C (2015). Deregulation of glyceraldehyde-3-phosphate dehydrogenase expression during tumor progression of human cutaneous melanoma. Anticancer Res.

[CR26] Schmittgen TD, Zakrajsek BA (2000). Effect of experimental treatment on housekeeping gene expression: validation by real-time, quantitative RT-PCR. J Biochem Biophys Methods.

[CR27] Shi Y, Felley-Bosco E, Marti TM, Orlowski K, Pruschy M, Stahel RA (2012). Starvation-induced activation of ATM/Chk2/p53 signaling sensitizes cancer cells to cisplatin. BMC Cancer.

[CR28] Sørby LA, Andersen SN, Bukholm IR, Jacobsen MB (2010). Evaluation of suitable reference genes for normalization of real-time reverse transcription PCR analysis in colon cancer. J Exp Clin Cancer Res.

[CR29] Tratwal J, Follin B, Ekblond A, Kastrup J, Haack-Sørensen M (2014). Identification of a common reference gene pair for qPCR in human mesenchymal stromal cells from different tissue sources treated with VEGF. BMC Mol Biol.

[CR30] Vaiphei ST, Keppen J, Nongrum S, Chaubey RC, Kma L, Sharan RN (2015). Evaluation of endogenous control gene(s) for gene expression studies in human blood exposed to 60Co γ-rays ex vivo. J Radiat Res.

[CR31] Vandesompele J, De Preter K, Pattyn F, Poppe B, Van Roy N, De Paepe A, Speleman F (2002). Accurate normalization of real-time quantitative RT-PCR data by geometric averaging of multiple internal control genes. Genome Biol.

[CR32] Vigelsø A, Dybboe R, Hansen CN, Dela F, Helge JW, Guadalupe Grau A (2015). GAPDH and β-actin protein decreases with aging, making Stain-Free technology a superior loading control in Western blotting of human skeletal muscle. J Appl Physiol.

